# A randomized controlled trial of an mHealth intervention for increasing access to HIV testing and care among young cisgender men and transgender women: the mLab App study protocol

**DOI:** 10.1186/s12889-021-12015-w

**Published:** 2021-10-29

**Authors:** Olivia R. Wood, Robert Garofalo, Lisa M. Kuhns, Thomas F. Scherr, Ana Paola Mata Zetina, Rafael Garibay Rodriguez, Nathanael Nash, Marbella Cervantes, Rebecca Schnall

**Affiliations:** 1 Division of Scholarship and Research, Columbia University School of Nursing, 516 W. 168th Street, New York, NY 10032 USA; 2 Robert H. Lurie Children’s Hospital of Chicago, 225 E. Chicago Avenue, Chicago, IL 60611 USA; 3 Vanderbilt University, 2201 West End Avenue, Nashville, TN 37235 USA

**Keywords:** HIV/AIDS, Mobile health, Randomized controlled trial, Sexual minority

## Abstract

**Background:**

The number of youth living with HIV in the United States (US) continues to rise, and racial, ethnic, and sexual minority youth including young men who have sex with men (YMSM) and young transgender women (YTGW) bear a disproportionate burden of the HIV epidemic. Due to social and healthcare system factors, many YMSM and YTGW do not seek HIV testing services and are therefore less likely to be aware that they are infected. Mobile health technology (mHealth) has the ability to increase uptake of HIV testing among these populations. Thus, the mLab App—which combines HIV prevention information with a mobile phone imaging feature for interpreting at-home HIV test results—was developed to improve testing rates and linkage to care among Black, Latino, and other YMSM and YTGW living in New York City and Chicago and their surrounding areas.

**Methods:**

This study is a three-arm randomized controlled trial among YMSM and YTGW aged 18–29 years. Participants are randomized to either the mLab App intervention including HIV home test kits and standard of preventive care, standard of preventive care only, or HIV home test kits and standard of preventive care only.

**Discussion:**

mHealth technology used for HIV prevention is capable of delivering interventions in real-time, which creates an opportunity to remotely reach users across the country to strengthen their HIV care continuum engagement and treatment outcomes. Specifically during the COVID-19 pandemic, mHealth technology combined with at-home testing may prove to be essential in increasing HIV testing rates, especially among populations at high-risk or without regular access to HIV testing.

**Trial registration:**

This trial was registered with Clinicaltrials.gov (NCT03803683) on January 14, 2019.

## Background

The number of youth living with HIV in the United States (US) continues to rise, and the epidemic is exacerbated in racial, ethnic, and sexual minority youth who bear a disproportionate burden of the HIV epidemic. Reports show that 69% of new infections occur in young men who have sex with men (YMSM) and young transgender women (YTGW), and the epidemic is further magnified in Black and Latino youth [1–12]. There are large disparities in HIV testing rates in youth and more specifically in our target study population, Latino and Black YMSM and YTGW. The low uptake of HIV testing in youth can be explained by a number of factors.

Developmentally, youth often perceive themselves to be at low-risk for acquiring HIV as well as having an inaccurate perception of its impact [12]. There are also social and healthcare system factors that make YMSM and YTGW more vulnerable to becoming infected with HIV and less likely to be tested for HIV. Social factors include stigma, homophobia, transphobia, and racism. These factors may cause YMSM and YTGW to feel rejected and isolated [9, 12–15], and as a result they may not disclose their sexual orientation or gender identities [16] or seek HIV prevention and testing services [12]. Healthcare system factors include limited and inadequate access to youth centered HIV testing services [9, 17]. As a result, many YMSM and YTGW do not seek HIV testing services and are therefore less likely to be aware that they are infected [9, 11, 16, 18]. HIV+ youth who do not know they are infected are therefore not engaged in lifesaving treatment and care, and are at risk of infecting others [9]. This highlights the urgent need for interventions to increase the uptake of HIV testing in Black and Latino YMSM and YTGW.

One such method of increasing uptake of HIV testing among these populations is the use of mobile health (mHealth) technology. Past interventions have found mHealth technology to be a powerful platform for the delivery of HIV prevention interventions, including HIV testing [19, 20], and especially relevant for racial and ethnic minority youth [21]. Approaches using mHealth have the advantage of a simple interface for users, accessibility anywhere cell signals/Wi-Fi are available, relative affordability, and the ability to reach stigmatized and disenfranchised populations [22, 23]. While preliminary evidence suggests that mHealth technology (e.g., smartphone apps) is feasible, engaging, and effective for promoting HIV prevention and care outcomes among youth, many apps have not been designed by end-users and, of those that do exist in the marketplace, none have been well evaluated with YMSM or YTGW specifically [24]. Furthermore, the need for evaluation is even greater in Black and Latino YMSM and YTGW due to socioeconomic factors, cultural norms, stigma, homophobia, transphobia, and discrimination [25–34].

Building on our preliminary work [35, 36], our multi-disciplinary team (public health scientists, clinicians, and engineers) developed the mLab App, an innovative mobile and connected technology that combines HIV prevention information with email and text notifications for testing with a mobile phone imaging feature and algorithm for interpreting the visual results of the OraQuick rapid home HIV self-test to provide accessible, objective, secure, and real-time feedback on HIV test results. The mLab App also contains an automated data collection and results reporting feature, which relays test results back to the research team and the study participant, triggering messages to encourage future repeat testing for those who receive a non-reactive test or linkage to confirmatory testing and treatment for those with a reactive test.

### Study objective

In response to the public health need for the development of efficacious interventions targeted at high-risk youth, our study aim is to test the mLab App’s ability to improve both HIV testing rates and linkage to care among Black, Latino, and other YMSM and YTGW living in New York City and Chicago and their surrounding areas. Our study aim was determined with the goals of the Ending the HIV Epidemic in the US (EHE) plan set forth by the US Department of Health and Human Services [37] and thus, is of particular importance to national HIV strategy. This manuscript describes the mLab App study protocol.

### Ethics and consent

All study procedures were reviewed and approved by the Columbia University and Ann & Robert H. Lurie Children’s Hospital of Chicago Institutional Review Boards. Study participants provide written informed consent and HIPAA authorization prior to enrollment. The mLab App is an FDA regulated device and has been assigned IDE #18348.

## Methods

### Design

This study is a three-arm randomized controlled trial among YMSM and YTGW aged 18–29 years. Participants are randomized to either the mLab App intervention, HIV home test kits, and standard of preventive care (Arm 1), standard of preventive care only (Arm 2), or HIV home test kits and standard of preventive care only (Arm 3). Regardless of randomization, all participants schedule 6-month and 12-month post-baseline visits with study staff as part of the intervention, with brief check-ins via text-message occurring at 2-months, 4-months, 8-months, and 10-months post-baseline as well. Participants in all arms will receive standard HIV testing and education at their baseline visits. The differences between the three study arms are illustrated in Table 1.

**Table 1:** Comparison of Study Arms

	mLab App (Arm 1)	Standard of care (Arm 2)	OraQuick tests (Arm 3)
OraQuick	x		x
HIV Home tests			
Standard of Care	x	x	x
HIV testing education			
Condoms	x	x	x
PrEP education	x	x	x
Access to mLab App testing reminders	x		
Real-time interpretation of OraQuick test results	x		

### Recruitment and eligibility

Participants are recruited using a multi-modal strategy. Targeted recruitment is conducted via Facebook, Instagram, and Grindr, which involves creating and promoting advertisements to target the study populations in the two study cities. Participants are also recruited through flyers and the promotion of the study with community partners (e.g., community-based organizations) by distributing study related information and contact information of research staff. Those recruited through targeted recruitment and posted flyers are directed to an online web survey via REDCap for eligibility screening.

Eligibility criteria include: (1) 18–29 years of age; (2) assigned male sex at birth of any current gender identification; (3) understand and read English; (4) sexually active and at risk for HIV infection per CDC guidance (e.g., YMSM or YTGW and recent anal sex with men);^107^ (5) smartphone ownership; and (6) self-report being HIV-negative or unknown status (verified via HIV testing at the enrollment visit). Participants are excluded if they have a known diagnosis of HIV or if the investigators determine that participation may be detrimental to the participant or to the study.

### Sample size calculation

The targeted enrollment is 525 participants (210, 210, and 105 in arms 1, 2, and 3, respectively). The power and sample size calculations are based on a primary comparison between HIV testing rates in the mLab App arm (Arm 1) and the standard of care arm (Arm 2). The effect size is based on findings from the FORTH trial [38] in which promotion of home-testing resulted in a two times increase in frequency of HIV testing in high-risk MSM and a nearly four times increase in non-recent testers compared with standard care, without reducing the frequency of facility-based HIV testing. We estimate a 40% HIV testing rate over 6 months for the control group and approximately 60% HIV testing rate for the intervention group. We are using a more conservative effect size by assuming 60% testing rate for the intervention as compared to 85% found in the FORTH RCT study [38]. This 20% difference is equivalent to a medium effect size. All power calculations are based on alpha = 0.05 and two-sided tests of the primary comparison and an attrition rate of 20% at 6 and 12 months.

### Randomization

After providing informed consent, participants are randomized to study arms in a 2:2:1 ratio of: arm 1 - mLab App (2), arm 2 - Standard of Care HIV information (2), and arm 3 - HIV home tests (1), stratified by study site (Chicago, NYC) [38]. To reduce opportunities for selection bias, a variable permuted randomization block design was used [38]. The advantage of the permuted block design is that treatment assignment is pre-determined before the trial begins and assignment remains static throughout the course of the trial [38]. Within each city stratum, the REDCap program randomly assigns each participant to the next treatment allocation from a random-permuted block randomized sequence.

### Description of the intervention: the mLab App

**Fig. 1:**
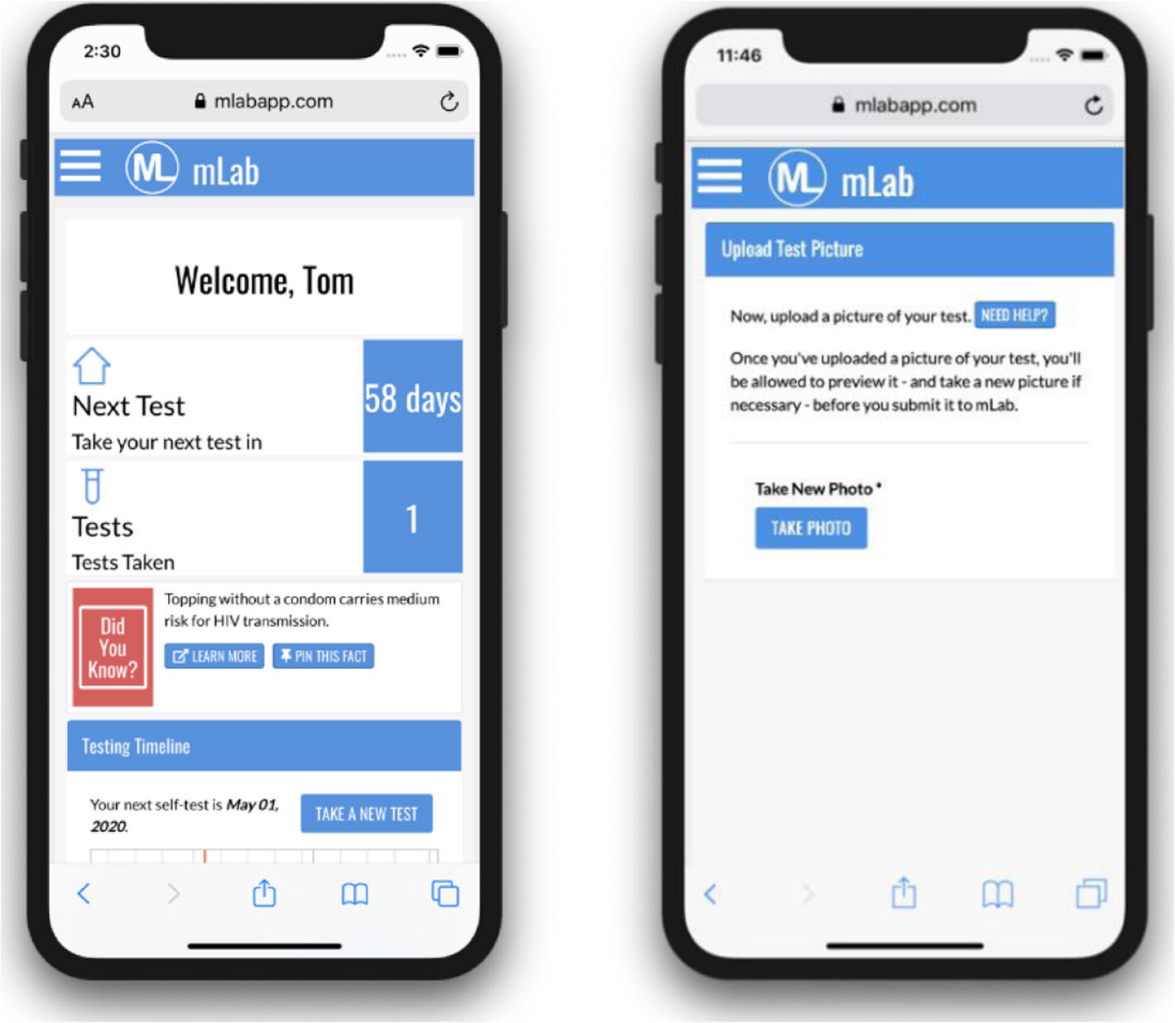
mLab App Home Screen

**Fig. 2:**
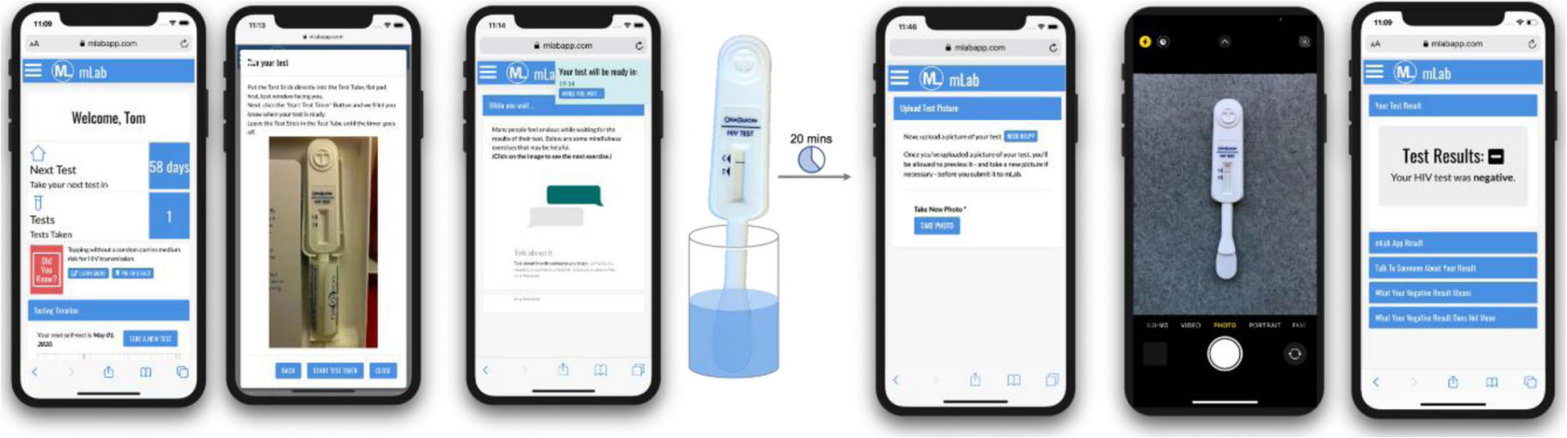
mLab App Testing Workflow

The mLab App is an FDA regulated investigational device (IDE #18348) that aims to test the ability of a diagnostic intervention delivered on a mobile platform to improve HIV testing and linkage to care among young adults at-risk for HIV. Because study participants can use the mLab App to analyze their test photos and interpret their results, FDA monitoring is required. The App was initially reviewed and approved by the Center for Biologics Evaluation and Research (CBER) at the FDA under specific requirements. Such requirements entailed including a labelling statement that the mLab App is an investigational device and the participant’s visual interpretation of the OraQuick test are the reference point for results. Participants access the mLab App using a login name and password that is assigned during their first visit. Once accessed, users are greeted by name in the app, can monitor their progress in the intervention on a movable timeline, and can read daily HIV prevention facts (Fig. 1). Most significantly, the app includes automatic notification reminders to complete HIV testing every 3-months. The automated image processing feature, where users can upload a photo of their completed OraQuick test, provides the (experimental) real-time test results (Fig. 2).

Following enrollment into the study, participants randomized to the intervention arm (arm 1) are provided with the mLab App and 2 OraQuick tests. Participants in arm 2 only receive the standard educational information (provided to all participants). Finally, those randomized to arm 3 receive 2 OraQuick tests and standard educational information. Participants in arms 1 and 3 receive an additional 2 OraQuick tests after their 6-month visit. At their baseline appointment, all participants regardless of arm are sent an email or text with links to mobile-optimized online prevention information, including PrEP and HIV testing information that is found on the CDC website. They will also receive a study information card listing the Columbia University School of Nursing/Lurie Children’s Hospital study teams’ contact information as well as condoms.

### Study assessments

Participants are enrolled in-person (before the onset of COVID-19) and remotely via Zoom videoconferencing with written electronic consent (after the onset of COVID-19 and for the duration of the study). After enrollment, participants complete study assessments at baseline as well as 6- and 12-month follow-ups via computer-assisted self-interviewing (CASI; either in-person or remotely via a web link). In total, there are three survey time points for all study participants (baseline, 6, and 12 months). Participants are required to show their ID and the face on their ID must match their face on the videoconferencing screen. Each participant is given a survey link matched to their study ID upon confirmation of their identity. All study data are securely stored at the primary study site in a limited access database by study ID. All hard copy participant information (e.g., study checklists, consent forms for in person enrollment) are securely stored at each study site in locked file cabinets with limited access.

### Primary outcome

The primary outcome is the proportion of participants who self-report being tested for HIV in the past 6 months. Beyond self-report, we also collect and analyze data on the app’s test interpretation performance. Participants in the intervention arm (arm 1) upload a photo image of their OraQuick test, enabling the study team to cross reference self-report with the visual results. We will assess and describe any bias in self-report of HIV testing in the intervention condition.

### Statistical analysis

Arms 1, 2, and 3 will be described with respect to baseline characteristics (e.g., means, standard deviations, ranges, and proportions). Before beginning formal analyses, we will examine the patterns of missing data, paying special attention to the balance of missing data in the study arms. We anticipate all participants will provide baseline data and approximately 80% will complete the 6- and 12-month post-enrollment assessment.

All multivariate analyses will be preceded by standard descriptive bivariate analyses for the key variables and relationships among them. These analyses will include means, frequency tables, histograms, and examination of distributions. Frequencies and rates of HIV tests, as well as corresponding confidence intervals, will be calculated for each arm. All statistical tests will be two-sided tests with the level of significance at 0.05. Hypotheses testing will be based on logistics models to compare HIV testing rates between the mLab App arm and the control arm. We will conduct stratified analyses to examine the differences in testing uptake in subgroups (i.e., racial/ethnic, age, YMSM vs. YTGW, and risk level). Effect sizes will be compared between arms 1 and 3 to describe differences in testing and linkage behavior attributable to the mLab App with at-home test distribution versus at-home test distribution only*.* Linkage to care will be measured by the percent of study participants who tested positive and attended a first HIV care appointment at NYP/Columbia or Lurie or who provide documentation of care at another clinic and the time linked to care after positive result. Follow-up surveys for HIV positive participants will also include questions regarding positive diagnosis, such as disclosure and seeking care. For participants who test HIV positive, we will compare the rates of linkage to care between the mLab App arm and the control arm using Fisher’s exact test. However, because the number of positive tests is expected to be approximately 25–40, this part of the study will not have power to detect a significant statistical difference between groups.

During use of the mLab App, participants submit images of their OraQuick self-test to the app for analysis by an automated image-processing algorithm. Prior to submitting this image for analysis, participants will be asked to interpret the results of the test using the instructions that are provided standard with the OraQuick test. This self-reported result will be recorded and uploaded with the user-submitted image of their rapid test. Study team members with expertise in rapid test use and analysis will evaluate the uploaded images of each rapid test and provide an additional assessment. The user-submitted result and the study team assessment will not be used by mLab in its automated image processing. All users that submit tests that are identified as positive, either by the automated image-processing in mLab, the study team personnel’s inspection of the image of the rapid test, or by the study participant themselves, will be referred for confirmatory testing.

Including the confirmatory test, which will be treated as the gold standard to determine whether a test result is truly false or positive, there will be results for reporting HIV testing results (1. User-submitted results, 2. Study team interpretation of uploaded images, 3. Automated mLab App results, 4. Confirmatory testing). The sensitivity and specificity for each of these methods will be compared using McNemar’s test. This will result in the following statistical comparisons for both sensitivity and specificity: 1) user-submitted analysis to study team analysis, 2) user submitted analysis to mLab analysis, 3) study team analysis of uploaded images, 4) user-submitted analysis to confirmatory testing, 5) study team analysis to confirmatory testing, 6) mLab analysis to confirmatory testing.

Multiple imputation (MI) methods will be applied to address missing values under the missing at random (MAR) assumption [38]. These analyses will be complemented with assessment of how sensitive the inferences are to the MAR assumptions. Sensitivity analysis will be performed based on selection models for dropout [38–41]. All analyses will use the ITT principle [42], which requires subjects’ data to be analyzed as randomized, regardless of whether they used the mLab App or not.

## Discussion

The expansion of smartphones and the significant growth in wireless technology has led to innovative methods of delivering health information to young people. In the United States, more than 99% of young adults between the ages of 18 and 29 have a cellphone and 96% are smartphone owners [43]. Due to the pervasive and universal presence of technology and its adoption by young adults, innovative mHealth technology has been a crucial approach to engage the youth in care including providing health information, support, and linkage to services [44]. The adaptability, convenience, and confidentiality of mHealth makes it appealing to young people seeking sensitive health information and care like HIV prevention and testing. Importantly, mHealth technology used for HIV prevention is capable of delivering interventions in real-time, which creates an opportunity to remotely reach users across the country to strengthen their HIV care continuum engagement and treatment outcomes.

As discussed, mHealth technology, specifically mobile medical apps (MMAs), are an integral part of health management for patients and providers. In 2017, more than 325,000 MMAs were available for download to smartphones, leading to an estimated 3.7 billion downloads among both patients and healthcare providers [45]. Because MMAs have become so ubiquitous today, regulation is necessary to ensure the safety and effectiveness of such technology, as well as the health and wellbeing of users. Specifically, the FDA monitors and regulates MMAs that are used in the treatment, prevention, mitigation, or diagnosis of a disease, which can include apps that track health metrics and provide patient-facing reminders for medication or testing adherence, like the mLab App does [46, 47]. FDA monitoring of the mLab App during this clinical trial will further inform the validity and reliability of the imaging algorithm used by the app.

Despite the expansion of MMAs, since March 2020, the COVID-19 pandemic has led to substantial challenges for acquiring healthcare services including HIV testing and linkage to care. Many sexual health clinics closed or suspended in-person operations in response to the pandemic’s “stay at home” orders [48]. The abrupt discontinuation of sexual health services threatened to undercut the work addressing rising HIV rates. The COVID-19 pandemic called for the fast implementation of new and easy-to-access methods to provide patients with HIV testing to avoid secondary epidemics [49]. HIV at-home self-testing, facilitated through the mLab App, is an innovative and reliable approach of accessing HIV tests and results without having to attend a clinic setting, where the risk of COVID-19 exposure and transmissions is high [50, 51]. The ease of use and accessibility has made home testing progressively popular [48]. Home testing provides ways to overcome testing barriers during a pandemic and reaches those at high-risk who rarely get tested.

Beyond the scope of the pandemic, the adoption of mLab App technology coupled with at-home testing has the potential to increase testing rates among populations at high-risk or without regular access to HIV testing due to economic or other limitations. At-home testing has been found to be acceptable among populations at risk for contracting HIV. One study found that, among those who had sought out testing in the past, 47% preferred at-home testing instead of testing in a clinical setting. The acceptability of at-home testing was reported to be 90% among those who were infrequent testers, highlighting the importance of at-home testing specifically for at-risk populations that may not have access to testing for a variety of reasons [52].

## Data Availability

N/A

## Abbreviations

CASI: Computer-Assisted Self-Interviewing
CBER: Center for Biologics Evaluation and Research
EHE: Ending the HIV Epidemic
IRB: Institutional Review Board
MAR: Missing At Random
mHealth: Mobile Health
MI: Multiple Imputation
MMA: Mobile Medical Apps
US: United States
YMSM: Young Men who have Sex with Men
YTGW: Young Transgender Women
